# A multi-task convolutional neural network for classification and segmentation of chronic venous disorders

**DOI:** 10.1038/s41598-022-27089-8

**Published:** 2023-01-14

**Authors:** Bruno Oliveira, Helena R. Torres, Pedro Morais, Fernando Veloso, António L. Baptista, Jaime C. Fonseca, João L. Vilaça

**Affiliations:** 1 Life and Health Sciences Research Institute (ICVS), School of Medicine, University of Minho, Campus de Gualtar, 4710-057 Braga, Portugal; 2 ICVS/3B’s - PT Government Associate Laboratory, Braga/Guimarães, Portugal; 3 Algoritmi Center, School of Engineering, University of Minho, Guimarães, Portugal; 4 2Ai – School of Technology, IPCA, Barcelos, Portugal; 5 LASI—Associate Laboratory of Intelligent Systems, 4800-058 Guimarães, Portugal; 6 Department of Mechanical Engineering, School of Engineering, University of Minho, Guimarães, Portugal

**Keywords:** Cardiovascular diseases, Biomedical engineering, Diagnosis, Disease prevention, Medical imaging

## Abstract

Chronic Venous Disorders (CVD) of the lower limbs are one of the most prevalent medical conditions, affecting 35% of adults in Europe and North America. Due to the exponential growth of the aging population and the worsening of CVD with age, it is expected that the healthcare costs and the resources needed for the treatment of CVD will increase in the coming years. The early diagnosis of CVD is fundamental in treatment planning, while the monitoring of its treatment is fundamental to assess a patient’s condition and quantify the evolution of CVD. However, correct diagnosis relies on a qualitative approach through visual recognition of the various venous disorders, being time-consuming and highly dependent on the physician’s expertise. In this paper, we propose a novel automatic strategy for the joint segmentation and classification of CVDs. The strategy relies on a multi-task deep learning network, denominated VENet, that simultaneously solves segmentation and classification tasks, exploiting the information of both tasks to increase learning efficiency, ultimately improving their performance. The proposed method was compared against state-of-the-art strategies in a dataset of 1376 CVD images. Experiments showed that the VENet achieved a classification performance of 96.4%, 96.4%, and 97.2% for accuracy, precision, and recall, respectively, and a segmentation performance of 75.4%, 76.7.0%, 76.7% for the Dice coefficient, precision, and recall, respectively. The joint formulation increased the robustness of both tasks when compared to the conventional classification or segmentation strategies, proving its added value, mainly for the segmentation of small lesions.

## Introduction

Chronic Venous Disorders (CVD) of the lower limbs are one of the most prevalent medical conditions in the adult population worldwide, representing 1–2% of the healthcare budgets in Western European countries and North America^1–3^. In the initial stages, symptoms associated with CVD include leg pain, discomfort, heaviness, and swelling. Due to the visual impact of this condition on the patient’s skin, it also results in low self-esteem, isolation, and depression, all of which impact the patient’s quality of life. As the disease worsens to varicose veins, edema, skin changes, and ulceration, the quality of life is reduced and the demand for treatment increases^2,4,5^. Due to the variety of signs and symptoms associated with CVD severity, correct diagnosis is essential to provide accurate treatment to the patients and to help in the management of medical resources^6–9^.

**Figure 1:**
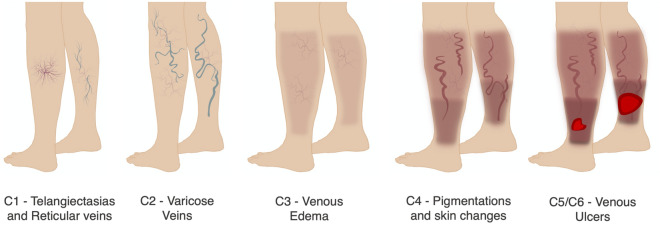
Chronic venous disorders (CVD) stages of development with respective severity classification (CEAP).

The signs of CVD are typically evaluated in terms of a structured clinical classification protocol named CEAP (Clinical, Etiologic, Anatomic, Pathophysiologic)^6,7^. This protocol incorporates a wide range of signs and symptoms of CVDs to describe their severity, ranging from C0 (no visible signs of venous disease) to C6 (active venous ulcer) (Fig. 1). While the CEAP scoring protocol is useful to classify the stages of CVD, it is relatively static, and thus, insufficient for a physician to determine quantitative changes in severity over time in response to the therapy^10,11^. Thus, the Venous Clinical Severity Scoring (VCSS) was proposed to supplement the CEAP protocol. The VCSS system evaluates 10 clinical characteristics scored from 0 to 3 (absent, mild, moderate, severe) to produce a 30-point scale of CVD severity that enables a more sensible evaluation of the treatment response^10,11^. Although both CEAP and VCSS protocols allow the physicians to report CVD diagnosis, infer the patient condition, and offer indicative information for the treatment, they require a physical examination and visual inspection of the patient’s skin to detect the occurrence and extent of all lesions^4,11^. Nevertheless, this process is challenging and is still totally dependent on the physician’s expertise, frequently resulting in incomplete recognition of all CVD signs and, consequently, undertreated CVDs^7,12^. Moreover, with the current scoring methods, a continuous quantification of the treatment evolution is still hampered.

The research on imaging tools for the examination of skin lesions has already demonstrated its clinical interest with increased diagnostic accuracy^13–16^. To help the diagnosis and monitoring treatment evolution of CVD, digital photographs are regularly captured and stored by healthcare professionals^17–19^. These photographs allow an easier evaluation of the patient’s condition by comparing the classification (i.e. according to CEAP and VCSS) and lesion extension in different periods^17,19,20^. References on the clinical images (e.g. ruler, taper) can also be used to ease the correlation with real-world units^17^. Nevertheless, skin images are difficult to classify and segment since the CVD lesions vary in shape and size^13,14^. To overcome similar medical problems, automated skin lesion segmentation and classification methods have been proposed in the literature. Among them, deep convolutional neural networks (DCNN) have achieved significantly improved performance in segmentation and classification tasks on skin images when compared with other image processing strategies^14,17^.

DCNN architectures such as VGGNet^21^, Fully Convolution Neural networks^22^, or U-Net^23^ architectures, proved to be effective in the diagnosis of skin lesions and reached predicted levels on par with dermatologists^14,24–27^. However, specifically for CVD images, few automatic strategies were still proposed, targeting mainly skin ulcer segmentation^17,20,28^, or CVD severity classification^29–31^. The interpretation of images with these skin lesions is particularly difficult. First, contrary to other medical images (e.g. CT, MRI) where standard and calibrated equipment is available, digital cameras are normally used by physicians to record photos of CVD lesions. Second, CVD presents a wide range of sizes and shapes, ranging from small vessels with 1 mm of caliber to varicose veins often protruding from the skin to skin ulcers with well-defined features. Interestingly, and although not validated in CVD, for high variable segmentation/classification situations, multi-task learning techniques have recently demonstrated their added value to empower network training^32–36^. These methodologies combine information from different tasks, boosting the generalization ability of the network, and showing improvement in the overall method’s performance. For CVD analysis, and in opposition to state-of-the-art skin lesion methods where both segmentation and classification are normally performed independently, this joint strategy could potentiate the results of each task while fulfilling the current requirements of normal clinical practice with both CVD classification and segmentation.

In this study, we propose a novel framework based on DCNN to segment CVD and classify its severity from medical images. Our proposed network, named as VENet, explores the advantages of multi-task learning by jointly learning both classification and segmentation tasks. This strategy is inspired by the U-Net architecture but redirects the high-level features from the deep stages into a new classification branch. Overall, this strategy has the potential to improve the accuracy of both tasks by sharing the same feature map and promoting each other during the training phase. VENet takes CVD images as input and outputs the CVD severity classification (according to CEAP) and segmentation results of the lesions from different severity levels, namely telangiectasias and reticular veins, varicose veins, and ulcers. For the development of the proposed framework, a new clinical dataset of CVD was constructed. The main contributions of this work are described as follows:

- A novel automatic methodology for segmenting and classifying CVD in clinical photographic images. To the best of our knowledge, this is the first attempt to perform simultaneously CVD lesions segmentation and classification;

- A new DCNN, VENet, for multi-class joint segmentation and classification of skin images in an end-to-end manner. With the joint learning of the classification and segmentation task, VENet has the potential to improve the robustness of both tasks;

- A validation of the proposed VENet pipeline against state-of-the-art single-task and multi-task segmentation and classification methods;

This paper is structured as follows. Section “Methods” describes the methodology of the proposed framework, with implementation details presented in Sect. “Implementation details”. Section “Experiments” introduces the validation experiments, followed by results in Sect. “Results”. In Sect. “Discussion”, the performance of each module of the proposed framework is discussed and compared with state-of-the-art results. The conclusions are given in Sect. “Conclusion”.

## Methods

### General overview

The proposed framework integrates classification and segmentation tasks in an end-to-end DCNN methodology. Initially, an RGB image is fed into the VENet, which produces two different outputs, namely a vector of probabilities and a set of segmentation masks for different CVD lesions. Specifically, the probability vector contains the severity level probability of the patient’s condition, according to 5 levels of severity: level 0—no visible signs of venous disease; level 1—telangiectasias /reticular veins; level 2—varicose veins; level 3—edema and skin changes including pigmentation and eczema; and level 4—healed/active venous ulcers (Fig. 1). Additionally, for each severity, a segmentation mask is generated with the pixel value corresponding to the probability of being a lesion with that severity.

**Figure 2:**
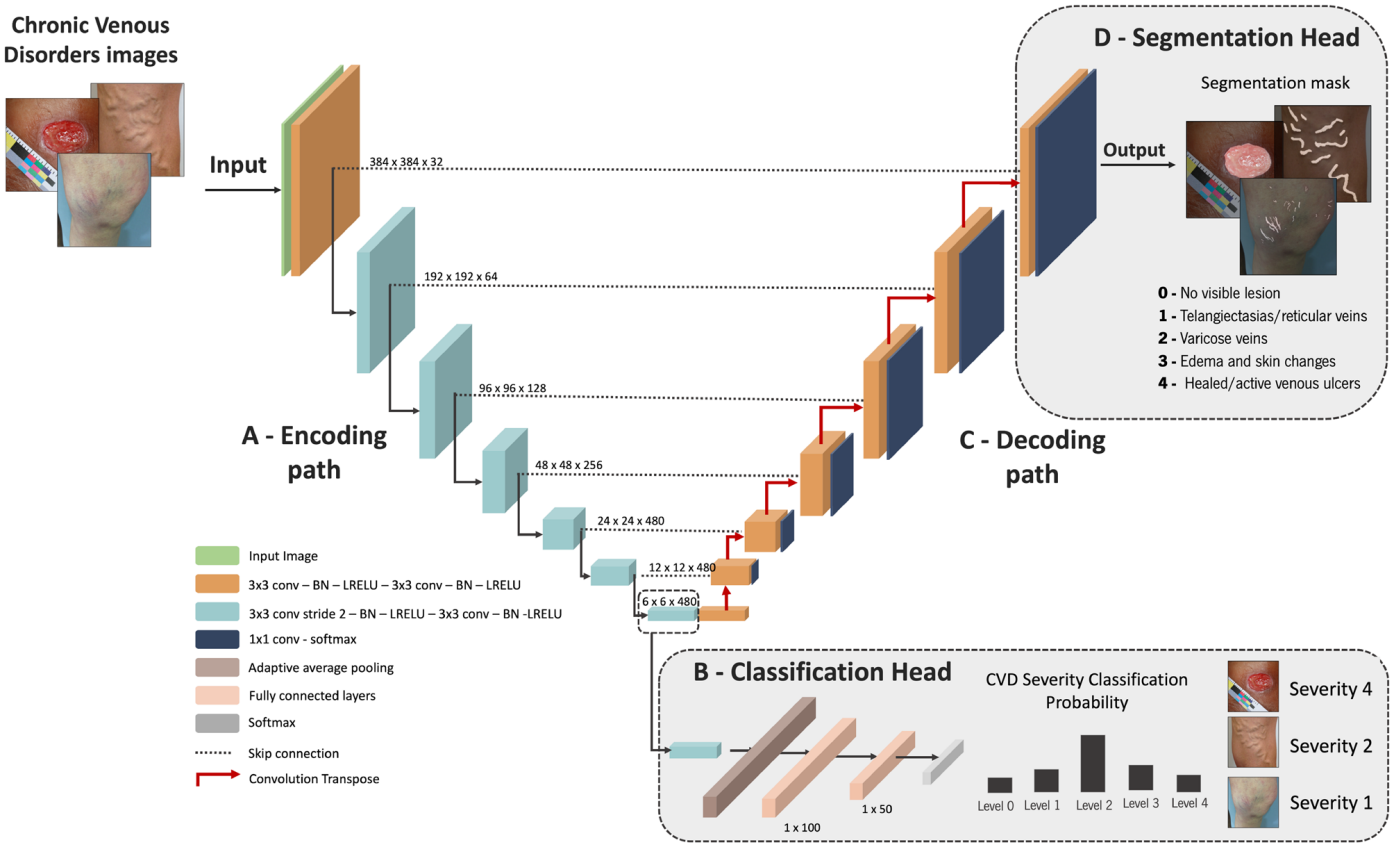
Overview of the proposed VENet for CVD severity classification and lesion segmentation. (**A**) Encoding path; (**B**) Classification head; (**C**) Decoding path; (**D**) Segmentation head.

The general overview of the proposed architecture is presented in Fig. 2. It is divided into four conceptual blocks. In the first (Sect. “Data augmentation”), data augmentation techniques are explored. This data is then fed to VENet, which architecture is fully explained in the second block (Sect. “VENet architecture”). Next, in the third block (Sect. “Loss function”), the multi-task loss developed to allow VENet to jointly learn the segmentation and classification tasks are described. Lastly, the post-processing strategy is presented in the fourth block (Sect. “Post-processing”).

### Data augmentation

To deal with CVD variability, DCNN-based approaches require a large amount of data to correctly perform a specific task^17,24^. However, access to a generalized medical dataset is challenging. Thus, to improve the generalization capacity of VENet and overcome overfitting problems, data augmentation was performed. In fact, data augmentation techniques have been shown to improve the efficacy of DCNN for the diagnosis of skin lesions^14,24^. Two different types of data augmentation were implemented, namely spatial-based (i.e. random flip, rotation, scaling, grid distortion, optical distortion, and elastic transformations) and pixel-based techniques (i.e. random gaussian noise, brightness, contrast, and gamma transformations). While spatial-based augmentation aims to deal with the variability of lesion shapes as well as the usage of different digital cameras, pixel-based augmentations are used to improve the robustness of the VENet concerning the acquisition conditions (e.g. lighting variability) and lesions’ appearance.

### VENet architecture

The augmented data is sent as the input of the VENet for CVD segmentation and classification. The VENet architecture utilizes the U-Net as the backbone, and it is divided into four parts, namely the encoding path, the classification head, the decoding path, and the segmentation head (Fig. 2).

#### Encoding path

The VENet encoding path is composed of downsampling blocks to extract high-level features. Each block of the encoding path is composed of two convolutional layers, with each layer consisting of a convolution, followed by batch normalization and a leaky rectified linear unit (Leaky ReLU). The downsampling is implemented using a strided convolution on the first layer of each encoding block. The initial number of feature maps is defined to be 32, which is double in each downsampling strided convolution operation. Moreover, to limit the computational cost of the model, the number of feature maps is limited to 480 (Fig. 2A). Note that, the generated features from the encoding path are shared by both classification and segmentation heads.

#### Classification head

For image classification tasks, state-of-the-art results are obtained using DCNN for high-level feature extraction followed by a classification head constituted with fully connected layers to get the final classification vector^25,37^. Here, the introduction of subsampling layers improves the efficacy of the network since it eliminates the redundancy of features, reducing overfitting and minimizing computational cost^22,38^. We propose to use the VENet encoding path to extract high-level features for CVD classification. Thus, a classification head is added to the bottom of the VENet. The high-level features are fed into the classification head composed of a convolution block, followed by an adaptive average pooling layer to allow VENet to deal with different input sizes. Finally, the features are fed into the final block composed of a fully connected layer, a Leaky ReLU activation function, another fully connected layer, and a softmax layer to get the diagnostic probability of each CVD severity level (Fig. 2B).

#### Decoding path

The VENet decoding path is constituted by upsampling blocks to restore the feature maps to the original input size, allowing the interpretation of low-level features. Each block of the decoding path is composed of two convolutional layers, a batch normalization, and a Leaky ReLU activation function. Between each decoding block, a subsampling operation is implemented as a convolution transposed. This makes the encoding and decoding path symmetric. To allow the propagation of spatial information, skip connections are established between the encoding path and the decoding path at the same level. To implement the skip connections, a concatenation of the feature maps of encoding blocks and decoding blocks is used (Fig. 2C)^23^.

#### Segmentation head

In the segmentation head of VENet, the feature maps from the last block of the decoding path are fed into three consecutive convolution layers, with each layer consisting of a convolution, followed by batch normalization and a Leaky ReLU. A softmax layer is used to obtain the final instance-level probability maps. The final number of probability maps is defined to match the number of CVD lesions categories. Moreover, to allow deep supervision of the training process, probability maps from each decoding block are generated using a convolution layer followed by a softmax layer. The deep supervision allows the incorporation of the gradients from the backpropagation process deeper into the VENet easing the training process^39,40^(Fig. 2D).

### Loss function

We use a multi-task loss with two parts, namely the classification and segmentation losses^41,42^. Similar to other classification frameworks^43,44^, we use multi-class cross-entropy for the classification loss. Thus, with $c \epsilon \left[\mathrm{1,2} \dots , C\right]$ being the class index and C representing the number of CVD severity classes, the classification loss, ${L}_{class}$, is given by:

1 $${L}_{class}\left({\varvec{y}},\widehat{{\varvec{y}}}\right)= -\sum_{c = 1}^{C}{y}_{c}\times \mathit{log}\left({\widehat{y}}_{c}\right),$$

where $y$ represents the one-hot ground truth label for each class $c$, and ${\widehat{y}}_{c}$ is the VENet softmax output of the classification head for the same class $c$.

For the segmentation task, we combine the cross-entropy loss with the DICE loss. Cross-entropy loss already showed to allow a smooth convergence, although being ineffective in the case of imbalanced data for segmentation (i.e. where the background is larger than the lesions). This limitation is minimized with the inclusion of DICE loss, which focuses on the shape similarity between the predicted mask and the ground truth CVD segmentation maps^39,41^. The DICE loss (${L}_{DICE}$) is described as:

2 $${L}_{DICE}\left({\varvec{g}},{\varvec{p}}\right)= -\frac{1}{C}\times \sum_{c = 1}^{C}\frac{2\times \sum_{i=1}^{I}{g}_{c}^{i}\times{p}_{c}^{i}}{\sum_{i=1}^{I}{g}_{c}^{i}+\sum_{i=1}^{I}{p}_{c}^{i}+\varepsilon } ,$$

where ${p}_{c}^{i}$ is the softmax output of the VENet segmentation head, ${g}_{c}^{i}$ the value of one hot encoding of the ground truth segmentation map for the pixel $i \epsilon \left[1, 2, ..., I\right]$, and class $c,$ with $I$ the number of pixels per image, and $\varepsilon$ a constant value to overcome division by 0. Thus, the segmentation loss, ${L}_{seg}$ is given by:

3 $${L}_{seg}= {L}_{DICE}+ { L}_{CE},$$

with ${L}_{CE}({\varvec{g}},{\varvec{p}})= -\sum_{c = 1}^{C}{g}_{c}^{i}\times \mathrm{log}\left({p}_{c}^{i}\right)$ being the CE loss for the segmentation task. Additionally, to enable the deep supervision of the training, the final segmentation loss is the weighted sum of the losses from all resolution outputs of VENet (*i.e.* from the decoding blocks and segmentation head), given by:

4 $${L}_{seg\_deep}= \sum_{r = 1}^{R}{{w}_{deep}}_{r}\times {{L}_{seg}}_{r},$$

where ${{w}_{deep}}_{r}$ is the weight for the softmax output of the $r$ VENet resolution, $r \epsilon [\mathrm{1,2}, ..., R],$ and $R$ is the number of outputs of the VENet. Thus, ${{\varvec{w}}}_{deep}=\left\{{w}_{{deep}_{1}\dots }, {w}_{{deep}_{R}}\right\}$ is the vector of weights for the deep segmentation supervision loss. Note that, since segmentation prediction masks of different stages have different sizes and cannot be compared directly to the ground truth segmentation map, downsampling of the ground truth segmentation for each level is performed. Finally, the multi-task loss function is defined as:

5 $${L}_{VENet}= {{\lambda }_{class}\times L}_{class}+ {{\lambda }_{seg}\times L}_{seg\_deep},$$

where ${\lambda }_{class},{\lambda }_{seg}$ is used to weigh each task loss. The minimization of the proposed multi-task loss during the learning problem described in Sect. “Implementation details” allows VENet to jointly learn to classify and segment CVD.

### Post-processing

From the output of the segmentation head of the VENet, the final predictions are obtained by selecting the label with maximum probability in each pixel. After, isolated segmented pixels are eliminated. In the end, a severity classification and lesion segmentation of the CVD images are obtained.

## Implementation details

All the images were initially resized to have a minimal side equal to 384 pixels, followed by a center crop to have the final size of [384 × 384]. Note that, the cropping operation was reviewed to guarantee that pixels corresponding to a lesion were not removed. After, normalization for the range [0, 1] was performed. For the spatial-based augmentation, the input images were randomly flipped, either horizontally, vertically or both, with a probability of 0.3. A rotation by an angle selected randomly from a Gaussian distribution in the range [− 180°, 180°] with a probability of 0.2, and an image scale selected randomly from a Gaussian distribution in the range [− 0.3, 0.3] with a probability of 0.2, was also applied. A grid distortion with a probability of 0.5 with distortion limits in the range [− 0.3, 0.3], an optical distortion with a probability of 1 with distortion limits in the range [− 0.3, 0.3], and a shift limit in the range [− 0.05, 0.05] were also used. Finally, elastic transformations with an alpha of 120º, sigma of 6, and alpha affine in the range [− 6, 6] were also implemented. For the pixel-based augmentations, Gaussian noise was added to the input image with a variance range of [0, 0.1] with a probability of 0.1. Random brightness and contrast augmentations selected randomly from factors in the range [− 0.25, 0.25] with a probability of 0.15 were also introduced. Finally, random gamma transformation with a probability of 0.3 selected from gamma limits in the range [70, 150] was also used. The augmentations were performed using the Albumentations framework^45^.

For the multi-task loss,${\lambda }_{class}$ and ${\lambda }_{seg}$ were defined to be 0.5 and 1, respectively, which showed to achieve stable performance. Moreover, the weights ${{\varvec{w}}}_{deep}$ are defined according to $w_{{deep_{r} }} = \,\left( {1/2r} \right)/\sum\nolimits_{i = 1}^{R} {1/2i}$ thus giving less importance to segmentation predictions of lower resolution. The training of VENet was performed simultaneously for both classification and segmentation tasks in 1000 epochs with a mini-batch size of 4 and using the Adam optimizer with an initial learning rate of 0.0001 and a learning rate decay following the “poly” learning rate policy ($lr=1- (1-epoch/\mathrm{max}\_epoch{)}^{power}$) with power defined to 0.9^46^.

## Experiments

### Data

**Figure 3:**
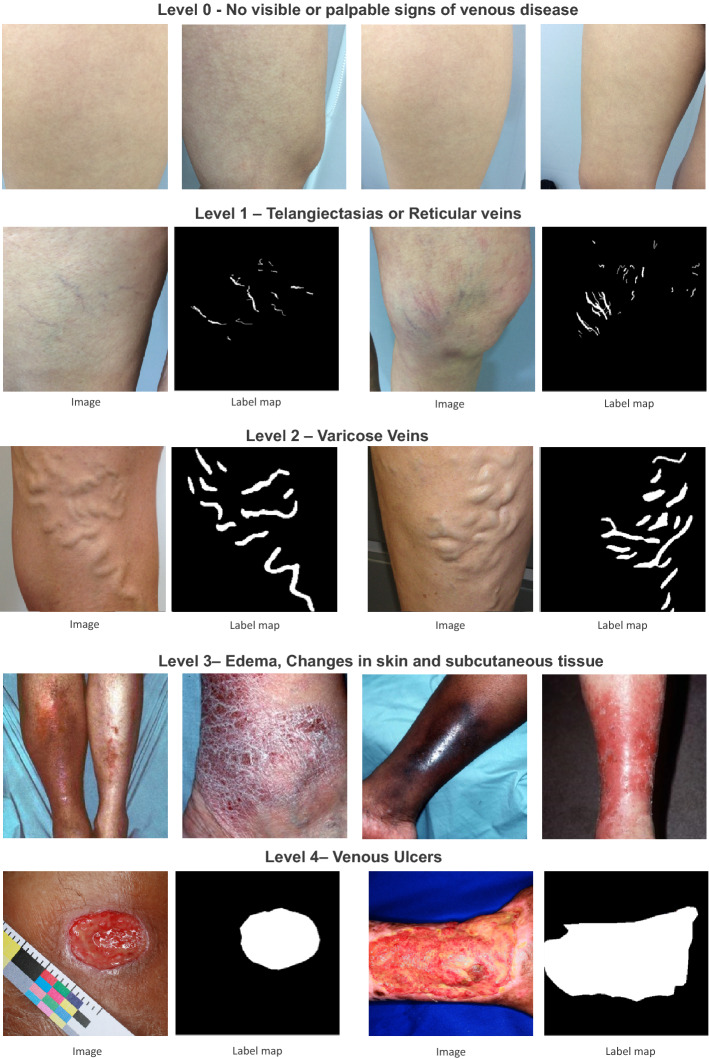
Examples of images used for the development of the proposed framework. Each row represents a different level of CVD severity. Note that, for severity levels with pixel annotations, two images are showed with the corresponding label map. For severity levels 0 and 3 with no manual annotations, four different images were presented.

A clinical database containing 1376 photographs of patients with CVD in lower limbs was constructed. The data was collected from normal clinical practice, where 522 images were obtained from two public datasets, namely 217 images from ULCER^47^ and 305 from the SD-198^48^. These images correspond to lesions from 5 different levels of CVD severity. All images were obtained using a digital camera. The distribution of images per severity level is described in Table 1. The included CVD images, excepting level 3 related images (i.e. lesions may cover the entire leg), contain pixel-level lesion annotations obtained by manual analysis of one experienced observer which were further validated by a licensed physician. Figure 3 illustrates examples of the different image sources and corresponding segmentations.

**Table 1:** Distribution of the dataset images per severity level.

Severity level	Ours	ULCER	SD-198	Total
0 – No visible or palpable signs of venous disease	223	0	0	223
1 – Telangiectasias or Reticular veins	237	0	0	237
2 – Varicose veins	127	0	0	127
3 – Edema, Pigmentation and other changes in skin and subcutaneous tissue	241	0	305	546
4 – Venous ulcers	26	217	0	243
TOTAL	854	217	305	1376

### Evaluation metrics

The proposed strategy was evaluated through the following metrics: Accuracy (ACC), Recall (REC), Precision (PRE), and Area Under the Curve (AUC) of Receiver Operating Characteristic (ROC). Moreover, for the classification, the balanced F1-score (F1-score) was used to evaluate the performance of the classification task. For the segmentation task evaluation, the Jaccard (JAC) and Dice coefficient (DICE) was also used to evaluate the similarity between the predicted and ground truth label map. Due to the multi-class segmentation and classification tasks, the average of the metric score from all classes was computed.

### Evaluation strategies

To evaluate the accuracy and robustness of the proposed VENet, several experiments were conducted, namely ablation studies (Sect. “Ablation studies”), a comparison against other conventional DCNN methodologies for lesion classification (Sect. “Classification DCNN”) and lesion segmentation tasks (Sect. “Segmentation DCNN”), and a comparison against other multi-task methodologies (Sect. “Multi-task DCNN”). All the networks were trained using the parameters described in Sect. “Implementation details”, except for the learning rate, which was adapted depending on the training loss curve of each network. The results were computed using Python code running on an Intel (R) i7 CPU at 2–8 GHz with 16 GB of RAM and an NVIDIA GTX 1070 GPU with 8 GB of memory and cuDNN 10.1 libraries. The proposed VENet and all the other conventional neural network architectures were implemented using the MONAI framework^49^ with the Pytorch library backend^50^. To measure the statistical significance of the classification results, a one-sided McNemar’s test in 2 × 2 tables (p < 0.05) was used. For the comparison of overall correctness, a joint 2 × 2 table was generated, which included all samples and showed the numbers of samples where none, one, or both methods produced a correct diagnosis. The statistical significance of the segmentation results of VENet was measured against all the other strategies in terms of DICE, PRE, and REC using a one-sided paired *t* test (p < 0.05). The D’Agostino-Pearson test was used to test the normality of the data. 

#### Ablation studies

Ablation experiments were performed to analyse the effect of the optimization approach, image augmentation techniques, loss function, deep supervision training, batch size, and the number of convolution kernels of VENet. First, the dataset was randomly divided into training, validation, and testing datasets, respectively 80%, 10%, and 10% of the initial dataset. Then, for each experiment, one component of the proposed method was replaced while keeping the remaining ones. Next, the modified VENet was trained and evaluated on the training and validation datasets, respectively. Note that, for the level 3 images (i.e. without mask), the segmentation loss component is neglected.

#### Classification DCNN

The performance of the EfficientNet-b4^51^, Resnet101^43^, DenseNet161^44^, and the encoding block of VENet (i.e. henceforward called VENetC) on the classification task was evaluated against our classification method with the multi-task approach. During these experiments, the dataset from the ablation studies mentioned above was used for training, while the testing dataset was used to obtain the final results.

#### Segmentation DCNN

The VENet’s segmentation performance was compared against the SegResNet^52^, DeepLabV3^53^, Fully Convolution Networks (FCN)^22^, and the VENet segmentation block only (i.e. henceforward called VENetS). The training of all networks was performed on the training dataset, while the results presented were computed on the testing dataset. Note that, for the level 3 images (i.e. without mask), the segmentation loss component is neglected during training.

#### Multi-task DCNN

The proposed VENet was compared against state-of-the-art multi-task learning methods in this experiment. Specifically, four different methodologies were tested, namely MTCSN^35^, DSI-Net^36^, Le et. al.^34^, and Che et al.^54^. All the multi-task frameworks were trained with a similar configuration to guarantee a fair comparison among methodologies. Note that, the convergence of all networks was manually verified. All results were measured on the testing database.

## Results

### Ablation studies

Table 2 depicts the method’s performance for the different methodological steps. Overall, the proposed strategy presented the best performance for all metrics, except for the PRE. The proposed method showed results in the test dataset of 78.3% DICE, with an ACC of 81.7% and REC of 77.6% for the segmentation task. Regarding the classification, an ACC of 97.8%, PRE of 97.6%, REC of 98.5%, and F1-score of 97.8% were obtained. The ADAM optimizer showed slightly superior performance than the SGD, while better results were obtained with the data augmentation strategies. Moreover, it is also shown that the introduction of deep supervision improved the VENet performance.

**Table 2:** Segmentation and classification performance of the ablation studies (mean).

Model	Segmentation				Classification			
	DICE	JAC	PRE	REC	ACC	PRE	REC	F1-score
Proposed	**78.3**	**67.0**	81.7	**77.6**	**97.8**	97.6	**98.5**	**97.8**
SGD	77.1	65.7	81.6	76.3	**97.8**	97.6	97.9	**97.8**
Initial conv = 16	74.1	62.4	82.1	72.3	97.1	96.7	97.5	97.1
Batch size = 2	67.3	54.8	81.6	63.5	89.8	89.6	87.4	89.9
Instance normalization	76.1	64.4	80.8	75.0	94.9	93.7	94.4	94.9
Loss seg. only DICE	69.8	58.0	85.0	65.2	97.1	**97.7**	96.6	97.1
Loss seg. only CE	65.0	53.0	89.4	56.7	94.9	95.3	95.0	94.9
Dropout 0.2	76.9	65.4	82.1	75.7	95.6	96.0	96.9	95.5
No contrast augmentation	77.4	66.1	82.3	75.9	95.6	96.3	95.9	95.6
No geometric augmentation	72.8	60.7	80.7	70.8	92.7	93.2	91.8	93.1
No loss deep supervision	75.4	63.5	80.9	73.5	94.9	95.6	94.5	95.0

*SGD* Stochastic gradient descendent optimizer with Nesterov momentum (μ = 0.95).

Loss Seg. only DICE – without cross entropy loss on Eq. (3)

Loss Seg. only CE – without dice loss on Eq. (3)

For each ablation experiment, one component of the proposed method was replaced or disable. For each row, the model column describes the component that was changed.

Bold values indicate best performance.

### Classification DCNN

**Figure 4:**
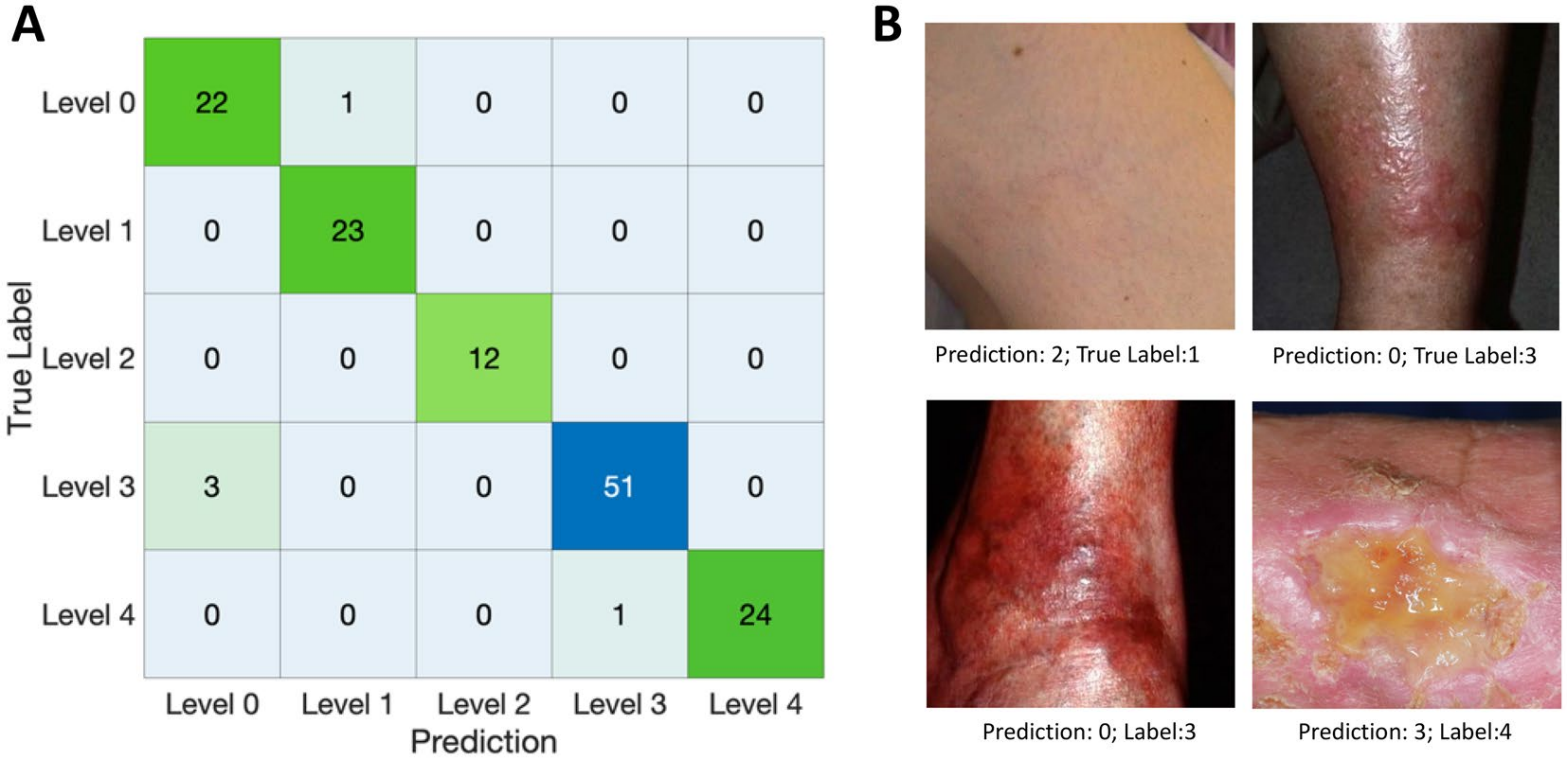
Confusion matrix of the proposed VENet for the classification of CVD images from the validation dataset. (**A**) Confusion matrix in each row represent the true label, while the column represents the VENet predicted classification. (**B**) Example pf wrong classified images by VENet.

**Figure 5:**
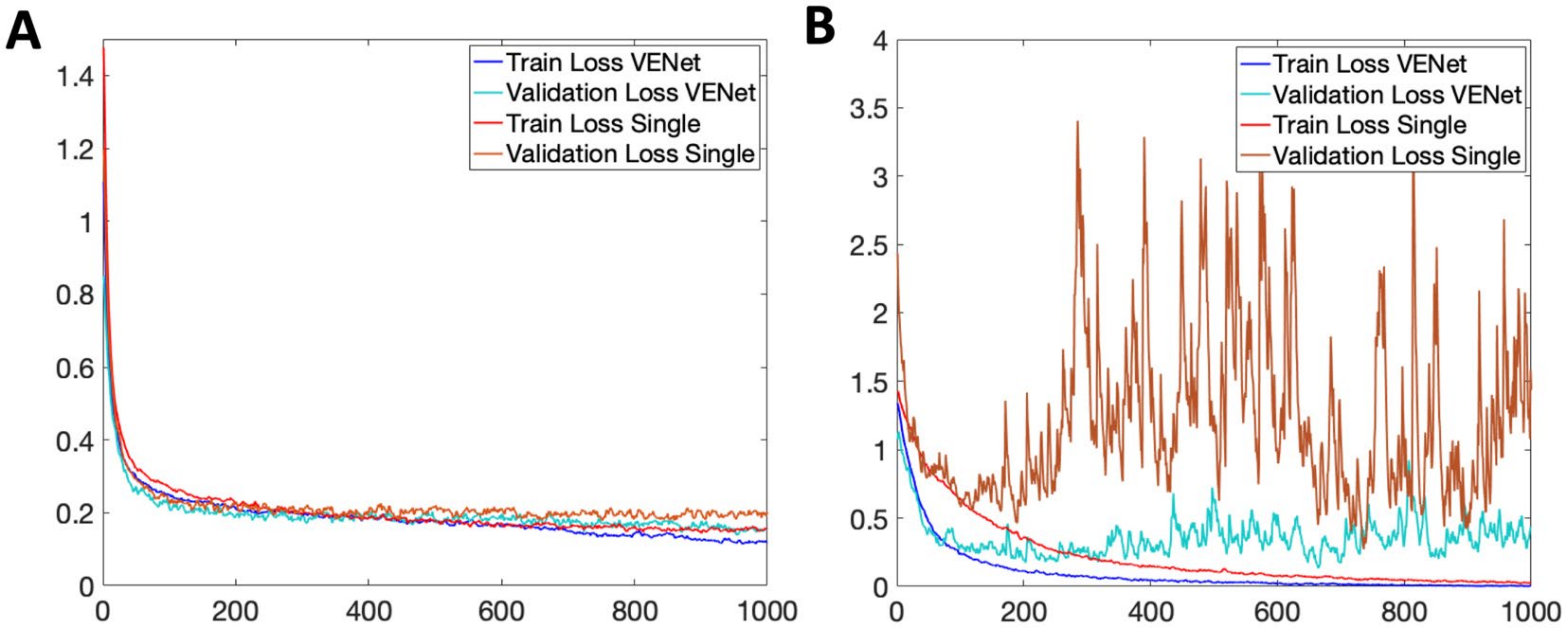
Comparison of loss curves of the training of single task DCNN (VENetS and VENetC for segmentation and classification, respectively) against the proposed multitask VENet. (**A**) Loss curves on the training/validation dataset for the segmentation task; (**B**) Loss curves on the training/validation dataset for the classification task. Note that, to ease interpretation, the segmentation loss curve was shifted up to have 0 as the minimum value.

Table 3 shows the comparison between the performance of the proposed VENet and other conventional DCNN architectures for the classification task. Generally, VENet presents the best average results against all the other networks, with an ACC, PRE, and REC of 96.4%, 96.4%, and 97.2% respectively. When comparing against VENetC, an improvement in the overall accuracy was shown. Still, no significant differences were observed between VENet and VENetC. Figure 4 shows the confusion matrix of VENet, showing that most of the classification errors originated from images with severity level 3. Figure 5B compares the loss curves of the training of single-task and multi-task networks for the classification task. VENet presented a lower loss value after convergence on both training and testing datasets.

**Table 3:** Comparision of the VENet performance against conventional DCNN for CVD classification task (mean ± S.D.).

Model	ACC	PRE	REC	F1-score	AUC
VENet	**96.4**	**96.4 ± 5**	**97.2 ± 2.6**	**96.3**	**99.6 ± 0.5**
VENetC	94.9	93.8 ± 5.8	96 ± 3.9	94.9	99.1 ± 0.8
DenseNet161	93.5^α^	93.3 ± 6.5^α^	92.5 ± 5.8^α^	93.5^α^	96.5 ± 5.1^α^
Resnet101	94.2	92.6 ± 8.5	95.4 ± 4.8	94.1	99.2 ± 0.7
EfficientNetB4	93.5^α^	93.3 ± 8.3^α^	93.5 ± 2.2^α^	93.4^α^	98.5 ± 1.1^α^

^α^p < 0.05. Two-sided McNemar’s test against the proposed VENet strategy.

Bold values indicate best performance.

### Segmentation DCNN

**Figure 6:**
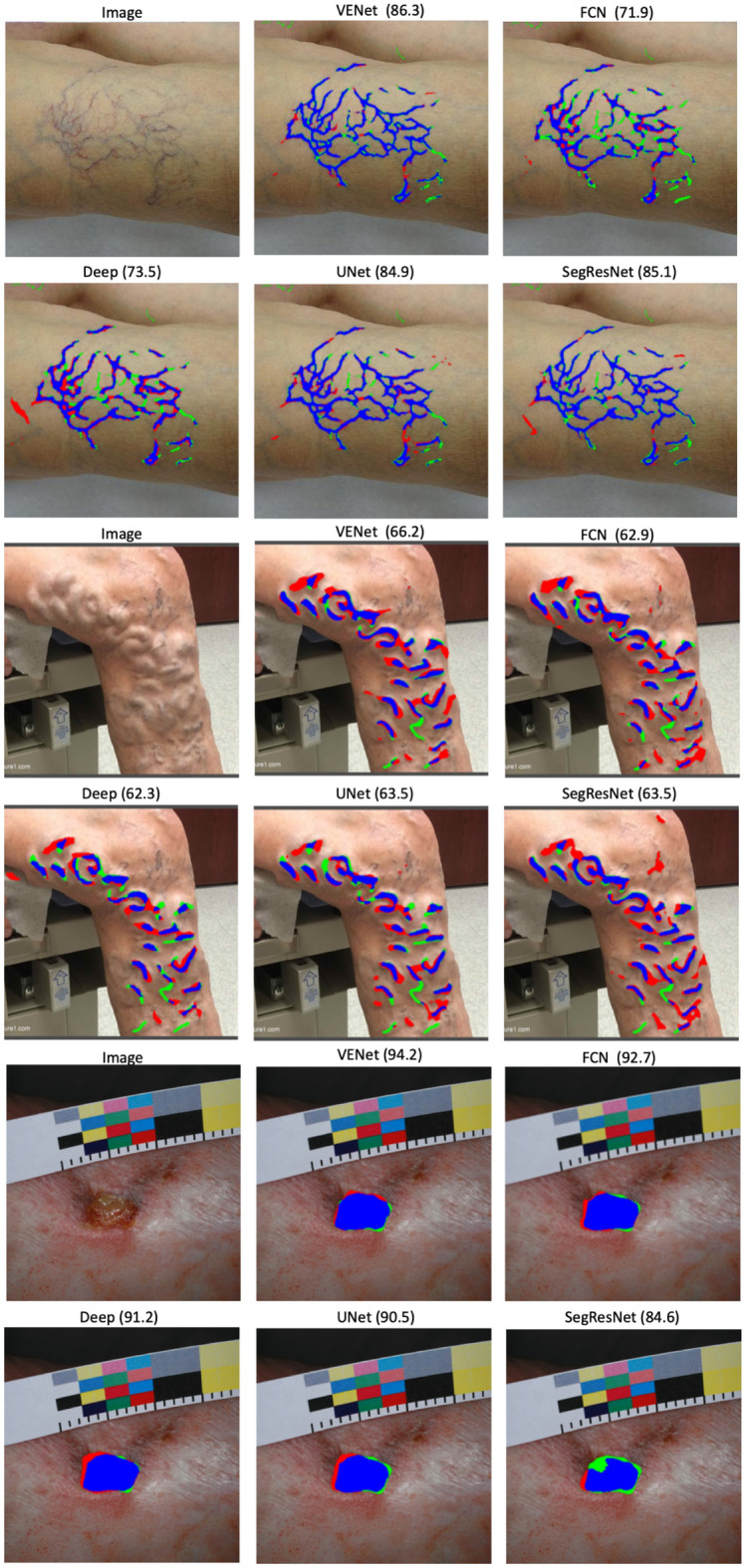
Segmentation examples of the proposed VENet and other conventional DCNN for the segmentation of CVD lesions, namely telangiectasias and reticular veins, varicose veins, and venous ulcers. The DICE metric is also provided for each segmentation result. The blue, red, and green colors represent the true positives, false positives, and false negatives, respectively.

Table 4 presents the results of the performance of the proposed VENet when compared against other conventional segmentation strategies. Overall, VENet showed the best performance in comparison with all the other evaluated strategies with an average DICE of 75.4%, a PRE of 76.7%, and a REC of 76.7%. The average DICE of VENet was 2.7% higher than the VENetS architecture. The segmentation results for each lesion separately show that VENet presents the best results against all the other DCNN architectures, with an average DICE of 70.8%, 62.8%, and 92.5% for the telangiectasias and reticular veins, varicose veins, and venous ulcers, respectively. Figure 5A compares the loss curves of the training of single-task and multi-task networks for the segmentation task. Again, analysing Fig. 5B, a better generalization ability of VENet against the single-task segmentation strategies was observed. Figure 6 shows example results for all the evaluated DCNNs.

**Table 4:** Comparison of the VENet performance against conventional DCNN for the CVD segmentation task.

Model	Telangiectasias reticular veins			Varicose veins			Venous ulcers			Overall		
	DICE	PRE	REC	DICE	PRE	REC	DICE	PRE	REC	DICE	PRE	REC
VENet	**70.8 ± 10.6**	70.3 ± 16.4	**75.3 ± 13.6**	**62.8 ± 12.1**	65.4 ± 8.9	63.1 ± 16.7	**92.5 ± 6.3**	94.4 ± 4.4	91.6 ± 9.9	**75.4 ± 9.7**	76.7 ± 9.9	**76.7 ± 13.4**
VENetS	68.1 ± 11.7^β^	72 ± 16.4	68.7 ± 16.2^β^	58.8 ± 16.9^β^	**67.4 ± 12.5**	55.8 ± 21.9^β^	91.3 ± 6.1	93.4 ± 6.2^β^	90.1 ± 9.7	72.7 ± 11.6^β^	**77.6 ± 11.7**	71.5 ± 15.9^β^
SegResnet	68.3 ± 11.5^β^	**73 ± 17.5**	69.8 ± 17.3^β^	62 ± 13.3	62.4 ± 10.6	**64.2 ± 19.6**	91.7 ± 8.3	92.8 ± 10.6	**91.8 ± 9**	74 ± 11^β^	76 ± 12.9	75.2 ± 15.3^β^
Deeplabv3	63.3 ± 12.2^β^	60.4 ± 15.7^β^	69.9 ± 13.7^β^	50.5 ± 24.1^β^	60.9 ± 9^β^	51.3 ± 25.7	88.6 ± 20	92.4 ± 4.1	86 ± 21.4	67.5 ± 18.8^β^	71.2 ± 9.6^β^	69.1 ± 20.2^β^
FCN	61 ± 12.9^β^	67.8 ± 15.5^β^	59.5 ± 17^β^	60.7 ± 10.2^β^	64.2 ± 8.1	60.4 ± 17.5	92.3 ± 6.6	**96.3 ± 3.5**^β^	89.4 ± 10.9^β^	71.3 ± 9.9^β^	76.1 ± 9	69.8 ± 15.1^β^

The results for the segmentation of each studied CVD lesion are presented separately. Moreover, the overall column presents the average of the individual lesion results (mean ± S.D.)

^β^p < 0.05, Paired *t* test against the proposed VENet strategy.

Bold values indicate best performance.

### Multi-task DCNN

Table 5 compares the performance of the VENet methodology against other state-of-the-art multi-task DCNNs. Overall, the VENet architecture presents the best performance for the segmentation of the CVD lesions, with an increment of 4% against DSI-Net, which achieved the second-best performance. Nevertheless, for the classification task, both DSI-Net and Che et al. frameworks achieved better performance with an accuracy of 97.8 and 97.1 against the 96.4 achieved by VENet.

**Table 5:** Comparison of the VENet performance against other multitask DCNN for CVD classification and segmentation (mean ± S.D.).

Model	Segmentation			Classification			
	DICE	PRE	REC	ACC	PRE	REC	F1-score
Proposed	**75.4 ± 9.6**	76.7 ± 10.1	**76.7 ± 13.3**	96.4	96.4 ± 5.0	97.2 ± 2.6	96.3
Le et al.	61.7 ± 11^β^	62.4 ± 12^β^	66.1 ± 16^β^	96.4	95.9 ± 5.2	97.1 ± 3.6	96.3
DSI-Net	71.4 ± 11.4^β^	**77.2 ± 10.8**	68.7 ± 15.7^β^	**97.8**	**97.5 ± 3.3**	**97.9 ± 3.4**	**97.8**
MTCSN	60.8 ± 14.6^β^	62.5 ± 14.4^β^	64.6 ± 21.2^β^	95.6	95.4 ± 3.5	95.9 ± 2.8	95.6
Che et al.	70.8 ± 10.1^β^	73.4 ± 10.7^β^	70.5 ± 13.8^β^	97.1	96.7 ± 3	97.5 ± 3.4	97.1

^β^p < 0.05, Paired *t* test against the proposed VENet strategy.

^α^p < 0.05, Two-sided McNemar’s test against the proposed VENet strategy.

Bold values indicate best performance.

## Discussion 

The proposed study presents a fully automated DCNN approach for severity classification and lesion segmentation from CVD images. Such a tool can potentiate diagnostic accuracy and enable the quantification of the treatment evolution. To the best of our knowledge, this is the first attempt to perform simultaneously CVD lesions segmentation and classification. Overall, the presented method showed high accuracy against state-of-the-art CVD lesion classification and segmentation techniques (i.e. single-task and multi-task). Indeed, the joint learning promoted the encoding path of VENet to extract more relevant features from the input images, leading to the generalization of the method, and ultimately resulting in increased robustness in both tasks. Specifically, for the segmentation, a statistically significant improvement in the performance was obtained, mainly for the segmentation of small lesions. For the classification task, although no statistical differences were measured, more stable training and an increase in classification robustness were also registered.

To validate the described pipeline, ablation analysis was performed. Table 2 proves the importance of each described component, achieving better results for most of the studied parameters of the base configuration, except for the PRE where isolated loss segmentation functions (i.e. only cross-entropy or DICE) showed superior performance. Indeed, cross-entropy is less susceptible to lesion size variations, allowing to have a more stable loss convergence during training^55^. Still, its low performance with imbalanced segmentation data also resulted in worse performance for the DICE metric. The introduction of data augmentation resulted in a significant impact on the performance of the VENet. Interestingly, geometric-based augmentation techniques proved to be more relevant for the final method’s performance when compared to contrast-based augmentation ones. The original dataset images were obtained from different sources and under different light conditions. This already introduces variability, which reduces the impact of contrast-based augmentation techniques on the method’s performance. On the contrary, venous disorders have high shape variability that is not represented in the original dataset. This limitation is reduced with the introduction of geometric-based data augmentation techniques. Note that, besides the deep supervision is focused on the decoding path, and consequently on the segmentation task of VENet, its absence resulted in a decrement in the accuracy of both tasks. This demonstrates that the joint learning of both tasks promoted the quality of the shared features extracted along the encoding path.

Table 3 validates the performance of VENet for the classification of CVD severity. Comparing both VENet and VENetC (i.e. encoding path plus classification head), although an improvement in the classification performance was achieved, no significant differences were found. Still, a better generalization ability of the VENet during training was verified (Fig. 5B). With the multi-task approach, the different tasks can provide regularization to each during the training phase^25,42^. Thus, the common features that lead to the optimized result of the VENet for both tasks are searched. This increases the robustness against the high variability between lesion types. Since the CVD lesions’ size are usually minor in the early stages, only a small region of the image can be occupied, which may lead the classification network to neglect them. Thus, by combining both tasks, the segmentation head can focus the classification network on the lesion’s spots, improving the overall robustness of the classification task during training.

Regarding the segmentation task, when looking individually for each lesion, VENet outperformed all the evaluated DCNN with an overall significantly better DICE and REC, with an improvement of approximately 2.5%, 4%, and 1.2% when comparing with VENetS for the segmentation of telangiectasias and reticular veins, varicose veins, and ulcers, respectively. Specifically, the second-best performance was obtained by SegResNet, suggesting that the incorporation of such architecture as the backbone of the multi-task framework could be an interesting solution. Thus, VENetS was ineffective in detecting equally good features among the different lesion types when compared with VENet. By assessing Fig. 6, it is possible to observe the advantages and consistency of the proposed methodology in the detection of all lesions. Altogether proved that the joint training of both tasks potentiate the generalization ability of the network, enabling the extraction of more relevant information from the CVD images. Thus, the benefit of jointly performing both classification and segmentation is demonstrated^32,33,41,56^. Note that, this benefit is stronger for the segmentation task than for the classification task where no statistical differences were found when compared against the single-task strategy. Comparing VENet against VEnetS per lesion performance, significantly better results were obtained for the segmentation of telangiectasias/reticular veins, and varicose veins. Thus, this suggests the superior performance of VENet for the segmentation of these smaller lesions.

Making a quantitative analysis of the results, an ACC of 96.4% with a PRE of 96.4% and REC of 97.2% was achieved for the classification of CVD severity, demonstrating the advantage of the described strategy for normal clinical practice. In Fig. 4, we can see that the images wrongly classified with severity 0 presented some artifacts related to the light condition which could have misled VENet. Most of the errors were from mislabelled images of severity level 3. This severity level comprises a wider range of lesions than the other ones, which seems to hamper the performance of VENet for the classification of these images. Moreover, the dataset showed an imbalanced nature between classes. Interestingly, the impact of using a class-balanced loss on VENet performance was also evaluated (for more details, the reader is kindly directed to the supplementary material, Appendix A). Although significant differences were not found, the incorporation of a class-balanced focal loss improved the classification results. Nevertheless, this also leads to a decrement in the segmentation performance, which can be the result of the optimization process starting to focus on minimizing specific classification errors that now have increased weight. Still, when the focus is on the performance of the lesion classification stage, class-balanced losses proved their added value. Note that, for the diagnosis of medical conditions, underdiagnoses have a higher cost for the patient’s condition than overdiagnosis. This feature can be learned by VENet by weighting mislabelled images of higher severity levels during the training process. With such a strategy, one is expected to reduce the underdiagnoses of CVD images.

For the segmentation task an overall DICE of 75.4%, with a PRE of 76.7% and a REC of 76.7% was achieved. Specifically, a superior average DICE was achieved for venous ulcers when compared with the results from other lesions. This result is explained by the larger dimensions with well-defined boundaries of the leg ulcers, making their segmentation simpler (Fig. 6). In opposition, vascular lesions such as telangiectasias are smaller lesions and difficult to detect. Varicose veins may not present well-defined boundaries since they are normally deeper and protruding vessels in the skin (i.e. the lesion is not illuminated evenly). This makes the segmentation task more challenging for these lesions. Here, post-processing techniques to fuse components of the same vessel, as proposed for other segmentation problems^57^, can be explored in the future to improve the method’s overall performance. Additionally, the segmentation result can notably increase the performance of the clinical diagnosis, enabling the quantification of treating evolution by simply comparing lesion masks of the patients over time. This gives enhanced information on the treatment monitoring to the physician and allowing to overcome the current limitations of the actual scoring strategies.

When comparing VENet against other multi-task frameworks, for the classification task, two networks presented slightly better results than VENet, namely DSI-Net and Che et. al. with an ACC of 97.8 and 97.1, against 96.4 of VENet. Still, no significant differences were obtained. For the segmentation task, a statistically significantly better performance of VENet was obtained, with an improvement of around 4% of the DICE for the second-best performance. This corroborates again the added value of VENet for the task of segmentation of CVD lesions.

Analysing the evaluated multi-task architectures, they all are similar with a backbone to extract features, followed by two heads for the segmentation and classification tasks. To take advantage of features from both tasks, and guarantee consistency in the prediction the DSI-Net, Che et al. and MTCSN networks proposed feature passing modules between the two branches. This may improve the segmentation performance for both DSI-Net and Che et. al. strategies when compared with the Le et al. network. Still, VENet outperformed the results of these networks. VENet also potentiates the interaction between the two tasks with feature-passing modules (i.e. concatenation) between the encoding and decoding paths. Nevertheless, instead of the evaluated multi-task DCNNs that only add such modules after the feature extraction, VENet establishes a connection between both tasks from the beginning of the encoding path. This enhances the propagation of spatial information by minimizing the loss of information during the down-sampling process, which may occur mainly for the smaller lesions, such as telangiectasias, reticular veins, and varicose veins. Indeed, VENet presented an average DICE of 70.8 and 62.8 against the 65.5 and 57.1 of the DSI-Net, for the segmentation of telangiectasias/reticular veins, and varicose veins, respectively. For the segmentation of venous ulcers, VENet presented an average DICE of 92.5 against 91.6 from the DSI-Net. As already shown, this proves the added value of the proposed VENet strategy, mainly for the segmentation of small structures.

Overall, the automatic VENet strategy can ease and fast the diagnosis of CVD lesions, being a relevant tool for physicians as well as a self-care module for patients, thus, potentially reducing the probability of underdiagnoses and promoting the treatment of CVD in the early stages. Notwithstanding, this study presents some limitations that could be addressed in the future. During training, the number of feature maps of VENet has been limited to 480 due to the computational cost. A half-precision training strategy can be explored in the future to minimize this limitation^58^. This may allow for increasing the number of feature maps as well as the batch size during training. Currently, VENet has around 34.15 million parameters, taking 25.8 ms for inference of a CVD image using the computational setup previously described in Sect. “Experiments”. Here, techniques to reduce memory and energy consumption could be explored, e.g. pruning^59^. Next, the results were obtained in a dataset of 1376 images, some obtained from the same clinical center. Therefore, specific intrinsic factors of the training dataset may be common, such as the camera acquisition parameters. Although data augmentation was explored to reduce this dependency, this raises concern about the method’s ability to generalize on a new test dataset with images from clinical centers not presented on the training/validation datasets.

Finally, CVD presents high levels of recurrence, which demands a periodic evaluation of the evolution of the patient’s condition^60–62^. For it, a computation of real-world units of all detected lesions is required, which can be accomplished with the introduction of a reference in the images (e.g. ruler, taper) or the usage of an RGB-Depth sensor. In fact, the impact of incorporating references, such as rulers or tapers on the images was tested in a preliminary experiment (please see the supplementary material, Appendix A). Although significant differences were not found for the DICE, the incorporation of references in the images also resulted in an increment in the performance of the VENet. Since they were mainly located on the severity level 4 images, they could be learned by DCNN to aid the classification and segmentation tasks. Note that, although the incorporation of such references has not been the focus of this work, this new development is envisioned as future work. With this extension, VENet can also enable a continuous quantification of patients’ conditions over CVD treatment and long-term recurrence of lesions. Indeed, the quantification of the treatment evolution is a desired feature among the treatment of all CVD severity levels, such as quantifying the treatment performance of vascular lasers for the treatment of telangiectasias (i.e. vessel clearance over treatment sessions), the recurrence of varicose veins after treatment (e.g. stripping, laser ablation), or also assess the evolution of healing of venous ulcers^17^. The computation of real-world lesion size using VENet work is envisioned as future work.

## Conclusion

In this work, we proposed a fully automated method for CVD severity classification and lesion segmentation. This method explores the multi-task learning advantages by learning both tasks simultaneously. The proposed VENet methodology allowed to boost the performance against other conventional DCNNs for the classification and segmentation tasks. While for the classification these resulted in increased robustness and training stability, although without statistical differences, for the segmentation task, statistically significantly better performance was obtained, mainly for the segmentation of small lesions. Overall, the proposed method can be explored in normal clinical practice to aid physicians and patients in the diagnosis/monitoring of CVD, overcoming underdiagnoses, and potentiating the treatment of these lesions in the early stages.

## Supplementary Information

Supplementary Information.

## Data Availability

The datasets generated and analysed during the current study can be made available at reasonable request to the corresponding author.
