# Supplementary material for: A multi-task convolutional neural network for classification and segmentation of chronic venous disorders
Source: Sci Rep. 2023 Jan 14;13:761. doi: 10.1038/s41598-022-27089-8 (PMC9840616; doi:10.1038/s41598-022-27089-8)
Supplement: Supplementary file 1 — Supplementary Information. [file 41598_2022_27089_MOESM1_ESM.pdf]

Appendix A – Analysis of the impact of a class-balanced loss and the incorporation of references on the performance of the VENet.

This document provides additional experiments on the VENet strategy. Specifically, two additional topics require special attention in this work.

First, as observed in Table 1 of the manuscript, the imbalanced nature of the dataset, namely for the images from CVD of levels 2 and 3. Thus, an additional ablation experiment was conducted to quantify the impact of a traditional class-balanced loss, and a class-balanced focal loss on the models' performance [1].

Second, the impact of incorporating references, such as rulers or tapers on the images. Although not addressed in this work, such incorporation eases the correlation with real-world units and increases the quantity of information in the image [2], [3]. Thus, an additional experiment was conducted to measure the impact of such incorporation on the performance of the model. Specifically, the VENet was trained and evaluated after pre-processing the dataset by removing all the references on the images (e.g. the ruler in Figure 3 of manuscript, bottom left). Both experiments were evaluated on the testing dataset.

To measure the statistical significance of the classification results, a two-sided McNemar's tests in 2x2 tables ($p < 0.05$) was used. For the comparison of overall correctness, a joint 2x2 table was generated, which included all samples and showed the numbers of samples where none, one or both methods produced a correct diagnosis. The statistical significance of the segmentation results of VENet was measured against all the other strategies in terms of DICE, PRE, and REC using a paired t-test ($p < 0.05$). The D'Agostino-Pearson test was used to test the normality of the data.

Table A1 compare the performance of the proposed VENet when trained under class-balanced losses and with images without references such as rulers and tapers. In general, VENet under class-balanced focal loss achieved better classification performance when

Table A1 – Segmentation and classification performance of the VENet under class-balanced losses and images without rulers and tapers (mean±S.D.)

Model	Segmentation			Classification			
	DICE	PRE	REC	ACC	PRE	REC	F1-score
Base	**75.4±9.6**	76.7±10.1	**76.7±13.3**	96.4	96.4±5.0	97.2±2.6	96.3
No Ruler	74±11	79.1±10.2$^{\beta}$	73±15.5$^{\beta}$	94.9	94.3±7.2	95.4±4.8	94.8
Focal	74.2±11.3	**80.1±10.3**$^{\beta}$	72±15.9$^{\beta}$	**98.5**	**98.4±3.2**	**99.3±1.5**	**98.5**
Weight	74.3±9.5	79±9.5$^{\beta}$	72.2±13.6$^{\beta}$	97.8	97.6±3.2	98.9±2.2	97.8

$^{\beta}$ $p < 0.05$, Paired t-test against the proposed VENet strategy.

$^{\alpha}$ $p < 0.05$, Two-sided McNemar's test against the proposed VENet strategy.

compared with the Base configuration. Nevertheless, a decrement in the segmentation performance was also observed. When trained VENet in images without references, a decrement in the segmentation and classification performance was also observed.

References

[1] Y. Cui, M. Jia, T.-Y. Lin, Y. Song, and S. Belongie, "Class-Balanced Loss Based on Effective Number of Samples." arXiv, Jan. 16, 2019. Accessed: Oct. 10, 2022. [Online]. Available: http://arxiv.org/abs/1901.05555

[2] D. Y. T. Chino, L. C. Scabora, M. T. Cazzolato, A. E. S. Jorge, C. Traina-Jr., and A. J. M. Traina, "Segmenting skin ulcers and measuring the wound area using deep convolutional networks," *Computer Methods and Programs in Biomedicine*, vol. 191, p. 105376, Jul. 2020, doi: 10.1016/j.cmpb.2020.105376.

[3] D. Y. T. Chino, L. C. Scabora, M. T. Cazzolato, A. E. S. Jorge, C. Traina, and A. J. M. Traina, "ICARUS: Retrieving Skin Ulcer Images through Bag-of-Signatures," in *2018 IEEE 31st International Symposium on Computer-Based Medical Systems (CBMS)*, Karlstad, Jun. 2018, pp. 82–87. doi: 10.1109/CBMS.2018.00022.
